# Evaluation of a regional ST-elevation myocardial infarction primary percutaneous coronary intervention program at the Rouge Valley Health System

**DOI:** 10.1186/1472-6963-14-449

**Published:** 2014-10-01

**Authors:** Pria MD Nippak, Jodie Pritchard, Robin Horodyski, Candace J Ikeda-Douglas, Winston W Isaac

**Affiliations:** Health Services Management Department, Ryerson University, 350 Victoria St, Toronto, ON M2K 5B3 Canada; Rouge Valley Health System, 580 Harwood Avenue South, Ajax, ON L1S 2J4 Canada

**Keywords:** Balloon, Myocardial infarction, Catheterization

## Abstract

**Background:**

ST-elevation myocardial infarction (STEMI) remains the second leading cause of death in Canada. Primary percutaneous coronary intervention (PCI) has been recognized as an effective method for treating STEMI. Improved access to primary PCI can be achieved through the implementation of regional PCI centres, which was the impetus for implementing the PCI program in an east Toronto hospital in 2009. As such, the purpose of this study was to measure the efficacy of this program regional expansion.

**Methods:**

A retrospective review of 101 patients diagnosed with STEMI from May to Sept 2010 was conducted. The average door-to-balloon time for these STEMI patients was calculated and the door-to-balloon times using different methods of arrival were analyzed. Method of arrival was by one of three ways: paramedic initiated referral; patient walk-ins to PCI centre emergency department; or transfer after walk-in to community hospital emergency department.

**Results:**

The study found that mean door-to balloon time for PCI was 112.5 minutes. When the door-to-balloon times were compared across the three arrival methods, patients who presented by paramedic-initiated referral had significantly shorter door-to-balloon times, (89.5 minutes) relative to those transferred (120.9 minutes) and those who walked into a PCI centre (126.7 minutes) (*p* = 0.047).

**Conclusions:**

The findings suggest that the partnership between the hospital and its EMS partners should be continued, and paramedic initiated referral should be expanded across Canada and EMS systems where feasible, as this level of coverage does not currently exist nationwide. Investments in regional centres of excellence and the creation of EMS partnerships are needed to enhance access to primary PCI.

## Background

Despite improved treatments, ST-elevation myocardial infarction (STEMI) remains the principle cause of death in developed countries [1]. Primary interventions used to treat STEMI in its acute phase are thrombolytic and percutaneous coronary intervention (PCI). While both are effective, research indicates a significant benefit to using PCI as the primary reperfusion therapy over fibrinolytics in acute myocardial infarction (MI) [2]. Furthermore, analysis of the multinational Global Registry of Acute Coronary Events (GRACE) has shown that an increasing use of primary PCI combined with decreased use of thrombolytics is associated with lower mortality [3]. Primary PCI has also improved short-term outcomes for patients over thrombolytics [4–6], resulting in less recurrent ischemia and angina, less cardiogenic shock, and a decreased length of stay [7–13].

A growing use of PCI has precipitated the development of specific guidelines by the American Heart Association (AHA) aimed at improving mortality rates and overall patient outcomes [13]. Time to PCI treatment, often referred to as the door-to-balloon time, has been identified as an important element that is influenced by several factors [14–18]. A ninety-minute door-to-balloon time has been set as the standard to achieve optimal benefits [14]. Several studies have shown that this door-to-balloon time is greatly influenced by the method of arrival to the PCI centre, with paramedic initiated referrals often meeting the standards for optimal outcomes [3, 15, 19–24]. Sierro et al., (2000) found paramedic initiated referral to be even more effective than hospital based strategies to reduce PCI door-to-balloon times [21]. However, given the infancy of these programs in Canada, few Canadian studies have contributed to this body of literature. Thus, a recent pilot expansion PCI initiative within the Central East Local Health Integration Network (CE LHIN) provided an opportunity to examine the role of method of arrival on the door-to-balloon times for STEMI patients receiving PCI [25].

This hospital’s interventional primary PCI program is considered to be a high volume program, which has been defined in the literature as performing more than 400 PCI procedures annually [26–28], and services 11% of Ontario’s population, which represents 1.5 million residents covering a large regional area [18, 25].

## Methods

### Setting

This East Toronto hospital is a community hospital that houses angiography and PCI services. The catchment area for the primary PCI site following expansion included: patients transported by Toronto emergency medical services (EMS) (in eastern Toronto areas) and Durham Region EMS; local patients from East Toronto who walk-in to the emergency department (ED); and patients transferred from The Scarborough Hospital campuses, and the Ajax-Pickering Hospital.

### Design

This was a retrospective review of patients with diagnosed STEMI who presented through one of three methods of arrival; either paramedic initiated referral, walk-in to the PCI centre ED, or transfer by EMS after walk-in to a community hospital ED without a PCI centre across the initial month expansion period from May to August 2010.

### Subjects

There were a total of 159 patients identified between the May to August study period. All patients that presented to a partner community hospital or the PCI centre ED, or were enrolled by paramedic-initiated referral and were STEMI diagnosed on initial electrocardiogram (ECG) and subsequently underwent primary PCI, were included in the study. The exclusion criteria were false positives (n = 41; Toronto EMS provider = 8; Durham EMS provider = 11; walk-ins = 11; transfers = 11), non-STEMI positive on initial presentation (n = 14) and those who were designated as in-patient in a participating hospital (n = 1). Two patients had incomplete data, which reduced the subject pool to 101. Ethics approval for the evaluation was obtained from the RVHS Research Studies and Research Ethics Board REB alongside the implementation of its “gold standard” pilot program [18].

### Procedure

For patients at the PCI centre ED diagnosed with STEMI, a code STEMI was activated. For non-PCI sites, the hospital staff immediately called 911 EMS for an urgent transfer to the PCI centre. The staff also called the PCI centre’s critical care unit (CCU) personnel who then alerted the primary PCI staff, and reserved a repatriation bed at their facility for the patient’s return in six hours. Once identification of a STEMI patient occurred, paramedics notified the CCU who then alerted the primary PCI centre team while en-route with the patient. Patients were considered stable as long as they had a secure airway and a systolic blood pressure greater than 80. If a patient arriving was unstable, then they were routed to the ED for stabilization before proceeding to the PCI laboratory, decreasing the time savings seen in direct PCI laboratory admission.

The door-to-balloon times were obtained from the monthly PCI reports, which contained information related to; the onset of symptoms (when available), and included information about the method of arrival, the time of first ECG, the arrival time to the catheterization laboratory, and the time EMS called.

The door-to-balloon start time for paramedic initiated referrals was designated as the time that the paramedic vehicle arrived at the address where the patient was transported from, which was documented within the ambulance call report. For both the walk-in and transfers from another hospital the door-to-balloon start time was the time when a patient was triaged at either the PCI centre’s ED (walk-ins) or one of the community hospital EDs (hospital transfers) before being transferred, which was reported in the patient chart. The end point door-to-balloon time for all cases was reported as the time the PCI balloon was inflated during the procedure, which was reported in the patient chart.

Also within the patient chart, the severity of heart failure using the Killip classification system [29] for each patient and whether the case was an “After Hours Case”, were recorded.

### Statistical analysis

Data from the monthly PCI reports were received in a detailed Excel sheet for the period of May 1, 2010 to August 30, 2010. Only cases where patients presented STEMI positive immediately upon first medical contact (triage at hospital or EMS) and were subsequently treated with primary PCI were selected.

Analysis was performed using SPSS 19.0.0 for Windows (SPSS Inc., 2010). A univariate analysis was used to compare door-to-balloon times for statistical significance, based on method of arrival. Chi-square tests were used to compare door-to-balloon times that met the <90-minute goal and those that exceeded the 90-minutes by method of arrival, as well as those that met or exceeded the 90-minute door-to-balloon time target by lab after-hours or regular-hours status.

## Results

Of the 101 patients who met the study criteria, 31arrived via paramedic initiated referral, 22 walked into the PCI centre ED and 48 were transferred from community hospitals after walk-in to their local ED.

**Figure 1:**
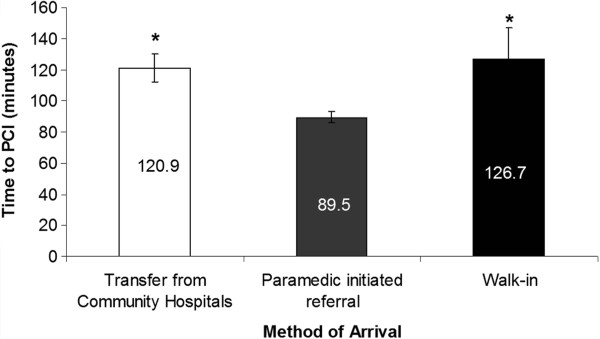
**Effects of method of arrival on door-to-balloon time for STEMI patients presenting to the primary PCI centre.** Paramedic-initiated referral had significantly shorter door-to-balloon times than either walk-ins or transfers from other hospitals. *p = 0.047.

Overall mean door-to-balloon time was 112.5 minutes (SD = 63.4). Patients who presented by paramedic initiated referral (89.5 minutes, SE = 62.08) had significantly shorter delays in door-to-balloon time when compared with patients who self-presented to hospital (126.7 minutes, SD = 62.07) and those who were transferred from community hospitals (120.9 minutes, SD = 62.09) (*p* = 0.047, *f* = 3.147, *df* = 2). Post hoc analysis using Fishers LSD the paramedic initiated referred group had significantly shorter door-to-balloon times compared to those who walked in to the community hospital ED (*p* = 0.034) (Figure 1) and those who were transferred (*p* = 0.030) from another hospital.

There was no statistically significant difference across the three methods of arrival with respect to meeting the 90-minute target versus exceeding the 90-minute target (χ2 = 2.38, *df* = 2, *p* = 0.304). When door-to-balloon times were compared in and outside of regular PCI lab hours 72% of primary PCI cases occurred after hours, while the rest occurred during regular lab hours. This did not statistically influence whether they met the 90-minute target or exceeded it, based on PCI lab after or during regular hours (χ2 = 2.59, *df* =1, p = 0.107).

When Killip class was examined, mean times varied in a non-linear fashion with Killip severity. Killip classes I through IV had means of 103.8 (SD = 13.1), 146.7 (SD = 32.3), 166.3 (SD = 61.3), and 137.3 (SD = 45.5) respectively. Killip class was not included in the analysis of door-to-balloon times by method of arrival because of the over representation of Killip class I and the incomplete data for all participants.

## Discussion

The paramedic-referred group had significantly shorter (89.5 minutes) door-to-balloon times relative to both the walk-in and transfer groups. These findings suggest that paramedic initiated referral of STEMI patients to a high-volume regional centre is an effective way of ensuring timely access to PCI services, which is consistent with the findings from other international studies [6, 10–12, 30, 31].

Paramedic initiated referral met the standard door-to-balloon time guidelines of 90 minutes set by the AHA [28] and was just slightly higher than the GRACE registry median time of 72–84 minutes [3]. The triage process, which was bypassed in the paramedic-initiated group, may have contributed to the longer door-to-balloon times in both the walk-in and transfer groups. For example, 20% of the patients in these two groups had triage times that were over 10 minutes. Similarly, another study indicated longer door-to-balloon times resulting from the hospital triage process [32]. These authors found the median door-to-balloon time to be 94 minutes, with 61% of field triaged patients experiencing door-to-balloon times of <90 minutes and only 36% of non-field triaged patients having door-to-balloon times <90 minutes [32]. Another study showed a 90-minute door-to-balloon time was met in 58% of paramedic direct triaged patients as compared to 38% of walk-in to emergency department patients, and 5% of patients transferred from other hospitals [24]. Finally, Dorsch et al., 2008 found that paramedic direct referral resulted in 94% of patients meeting door-to-balloon time standards vs. 29% of patients through the emergency department [19].

It is possible that some walk-in patients were asymptomatic upon arrival or displayed atypical symptoms thereby delaying the time to ECG and effective identification, relative to the paramedic initiated referral group who would have bypassed this triage process and had an ECG done in the field. Studies suggest that careful training of the emergency medicine physician, cardiology physicians and staff prior to implementation of a PCI centre can assist with effectively identify patients requiring PCI and thereby improve door to balloon times [33, 34]. More recently, patient demographics and procedural variations between hospitals have been shown to delay the triage process and contribute to longer door to balloon times [33, 35].

While the reported mean door-to-balloon time for the EMS group was considerably lower than the other two methods of arrival within the current study, the timeframe remains consistent with what has been reported within other studies [16, 23, 24]. In the current study there were no significant differences in the door-to-balloon times between the walk-in to PCI centre ED and the transferred to PCI centre group.

When methods of arrival on door-to-balloon times meeting or exceeding the 90 minute target were compared, no significant differences were found. These findings contradict other studies that have shown that paramedic referred patients tend to meet the <90 minute door-to-balloon time goal more often relative to other methods of arrival [9, 24, 36]. The absence of an effect however may be linked to the small sample sizes for each method of arrival, particularly within the paramedic initiated group where many of the reported times were just a few minutes over the 90-minute target. Also, the mean reported door-to-balloon time for the EMS group, which fell just under the 90-minute target, combined with the small variability in the mean door-to-balloon time, suggests that with a larger sample size it would be quite possible to replicate the findings in previous studies.

Seventy-two percent of primary PCI cases occurred after hours however there was no significant difference between door-to-balloon times based on PCI laboratory hours. These findings also differ from other studies that have shown longer door-to-balloon times after hours [19, 37–40]. Given the small sample size of cases within the laboratory hours no conclusion can be drawn.

### Limitations

This study was conducted in a high volume PCI centre with high volume operators. As such the findings may not be transferrable to centres that do not meet the AHA criteria as high volume centres [28]. As well, generalizations are limited given the sample size, the specific subgroups and any geographical issues that may be linked to the regional PCI centre.

Another limitation was that the “EMS on Scene” time used within the current study as the start time for the door-to-balloon time was the time the ambulance arrived at the patient location and not the time when the paramedics made contact with the patient. Using the time of initial patient contact as the start time would be more accurate.

Lastly, the number of false positives cases was slightly higher within one of the paramedic-referred groups relative to the other paramedic initiated group. One study suggests a PCI facility should expect a false positive rate of approximately 5% [41]. In the current study, one service provider had this expected false positive rate, however the second service provider displayed an almost 7% false positive rate over the other provider, but was consistent with the false positive rate found for both walk-ins and transfers. The underlying cause of this higher false positive rate in the one EMS provider group was found to be due to differences in the guidelines being applied between the two different EMS providers.

## Conclusions

The findings of this study suggest that continued partnership with EMS to promote improved communication would be beneficial to promoting timely door-to-balloon times in a high volume PCI centre [8, 28]. Refinement of EMS provider guidelines paired with more sensitive start time measures may support the development of a nationwide adoption of paramedic-initiated referral to PCI. Increased investments in regional PCI centres of excellence, and systems of rapid transfer from non-PCI facilities, paired with hospital specific improvements where catheterization staff and cardiologists are readily available and in close communication with the emergency department [42], could together serve to improve overall door-to-balloon times for PCI thereby improving patient outcomes. In conjunction with this, public education on the importance of accessing paramedic-initiated referral should occur.

In order to assess the ongoing impact of regional primary PCI centres, access to real time primary PCI databases need to be established to allow for the tracking of a variety of indicators and outcome measures. This would be ideally available on a provincial level, through e-health records, as patients are treated at multiple facilities. A comprehensive e-health record would allow for the capture of all relevant data, which could be used to improve patient care.

## Consent

Written informed consent was obtained from the patient for the publication of this report and any accompanying images.

## Abbreviations

AHA: American Heart Association
CCU: Critical Care Unit
CE LHIN: Central East Local Health Integration Network
ECG: Electrocardiogram
ED: Emergency Department
EMS: Emergency Medical Services
GRACE: Global Registry of Acute Coronary Events
MI: Myocardial infarction
PCI: Primary percutaneous intervention
STEMI: ST-Elevation Myocardial Infarction.
